# A case for inclusion of disordered Non-Death Interpersonal Grief as an official diagnosis: rationale, challenges and opportunities

**DOI:** 10.3389/fpsyt.2023.1300565

**Published:** 2023-12-14

**Authors:** Alexander Manevich, Einat Yehene, Simon Shimshon Rubin

**Affiliations:** ^1^Department of Psychology, Kinneret Academic College, Tzemach, Israel; ^2^Department of Behavioral Sciences, Kinneret Academic College, Tzemach, Israel; ^3^International Laboratory for the Study of Loss, Bereavement and Human Resilience and the School of Psychological Sciences, University of Haifa, Haifa, Israel; ^4^School of Behavioral Sciences, Academic College Tel Aviv-Yaffo, Tel Aviv, Israel; ^5^Department of Psychology, Max Stern Academic College of Emek Yezreel, Jezreel Valley, Israel

**Keywords:** loss, bereavement, Prolonged Grief Disorder, Diagnostic and Statistical Manual of Mental Disorders, International Classification of Disease

## Introduction

Grief and mourning are universal responses to the death of a significant other. Nevertheless, there are situations that diverge from the normative course of grief such that the intensity and duration of the responses are highly maladaptive and may require professional intervention. In the latest editions of the Diagnostic and Statistical Manual of Mental Disorders (1) and the International Classification of Diseases (2), a diagnosis of Prolonged Grief Disorder (PGD) was included. Two key conditions are required: (a) the diagnosis is given following the death of a significant other and (b) the grieving person experiences clinically relevant emotional and functional disturbances over an extended period of time.

This decision was made after a rich and continuous discussion, as well as quite a few controversies over the past decades surrounding the potential benefits and risks involved in including this type of diagnosis (3–8). Despite the official recognition of PGD, other disorders in the mourning process were not included in the diagnostic classification literature. These include grief reactions: (a) where the time since death is shorter than the defined criteria or the clinical picture does not meet the minimum threshold required for giving the diagnosis; and (b) stemming from losses which are not the result of an actual death.

Following the decisions to include PGD as a clinical diagnosis, we believe that conditions have been met to consider the broader class of maladaptive grief responses in non-death circumstances. In these cases, the prolonged grief reaction may be inherently present even though there has not been a death event. This would be the case where the mourning process is focused on a loved one, who is albeit still alive, but whose life functioning and personal identity are dramatically changed for the worse. These present the family-members with the loss of the person they knew and loved. Here too, there can be significant emotional pain and distress of grieving alongside distinct functional impairment in this grief.

Therefore, the purpose of the current paper is to present a framework to address these cases of non-death maladaptive grief reactions and the circumstances in which they arise. To do so, we briefly review salient relevant literature and consider some of the empirical evidence that has accumulated to date regarding a number of these conditions. To illustrate concisely, we will focus on caregivers of people living with a Major Neurocognitive Disorder (i.e., dementia) as a representative example with a rich data base. Next, we will lay-out several of the main dilemmas and arguments relevant to the inclusion of an official diagnosis of this type of grief disorder. Finally, we will conclude by presenting our position and suggestions for advancing discussion of these issues.

## Non-Death Interpersonal Grief

### Theoretical and clinical background of the concept

The theoretical and clinical origins of the phenomenon of grief that was not isomorphic with death lie in the writings of Lindemann (9) who coined the concept of “anticipatory grief.” Over the years, this concept has been further developed by Rando (10) and others (11, 12), and the idea remain that this is in response to the impending death of a significant-other as a result of a terminal-illness, such as untreatable malignancy.

Placing anticipatory grief as a response to the “dying” of a loved one, it made intuitive and clinical sense, to see anticipatory grief as part of the adaptive mourning for the dying patient. By spreading out the grief and mourning, the assumption that was widely accepted in the professional field was that the experience of grief before the expected death of a loved one is an adaptive response that will make coping at the time of death more bearable (13). However, it was found that this assumption was not sufficiently evidence based. In time, it emerged that high and prolonged levels of pre-death grief tend to predict adjustment difficulties during the bereavement period (13, 14).

Notably, past literature has well demonstrated that such non-death grief occurs not only in situations where death is indeed, but also in chronic situations that are not necessarily life-threatening, such as a personality change due to serious mental illnesses, traumatic brain injury, stroke, persistent disturbance of consciousness, and so on (15). Therefore, alongside the development of the anticipatory grief concept, other concepts were developed that referred to grief processes that occur while the person is still alive, such as non-finite loss (16), living loss (17), chronic sorrow (18), ambiguous loss (19), disenfranchised grief (20), and non-death loss (21).

“Non-finite loss” (16) is a term advanced to describe a loss triggered by a negative life event, which is long-lasting and where the source of the psychological or physical loss remains present and without a clear conclusion; “living loss” (17) also refers to ongoing, non-final grief experiences that are often found in non-death situations of significant loss. These losses remain part of the individual's life and require ongoing adaptation, characterized by their enduring presence and a distinct lack of finality; “chronic sorrow” (18) describes the long-term periodic sadness that individuals may experience in reaction to non-finite and living losses; “ambiguous loss” (19) refers to losses without a clear-cut death of a loved one and where there is significant blurring of the boundaries of the loss situation. The sense of loss and sadness can be manifest when the emotional connection to a loved one whose physical presence remains but where the psychological personhood is absent, or conversely, when the emotional connection remains but physical connection is not possible for an unclear duration; “disenfranchised grief” (20) refers to types of grief that pertain to losses and bereavements that lack social recognition or support, and where the mourner's grief is not acknowledged; and “non-death loss” (21) encompasses a wide array of life experiences of loss without reference to death.

However, despite the varying contexts and difference, a commonality emerges. People often grieve their loved-ones across a spectrum of situations that do not necessarily involve actual or immediate death. In some cases, these situations will last for years. In light of this, for our current discussion, we will employ a concept that encapsulates this broad range of losses involving a living loved-one: Non-Death Interpersonal Grief (NDIG). These losses may be accompanied by complex circumstances documented in the research literature as significant risk factors for high levels of NDIG. The aforementioned include the lack of recognition, legitimacy and social-support in the grieving process (22), as well as ambiguity and lack of clarity in relation to the loss. These conditions hinder adaptation and accommodation to the current realities of these losses resulting in ongoing grief which may remain highly elevated and debilitating (23). In addition, the presence of traumatic elements in the caregiving circumstances contribute to complications such as can be seen in caring for loved-ones with serious neuropsychiatric behavioral manifestations as in some of the dementias (24), etc.

Given space constraints, we will succinctly concentrate on grief associated with caregiving in Alzheimer's Disease and Alzheimer's Disease Related Dementias (AD/ADRD). This serves as a representative example to illuminate the issue at hand and its significance. The selection of AD/ADRD is informed by both the abundant research available to date and the profound impact of the disease on affected individuals, their informal caregivers, and society in general.

### NDIG in the context of AD/ADRD as a case study

AD/ADRD includes a range of major neurocognitive disorders with distinct origins and clinical manifestations. It is a progressive syndrome characterized by cognitive and functional impairments, which generally co-occur with negative changes in personality and behavior (1). Experts estimate that the number of people living with AD/ADRD is expected to increase from 57.4 million cases globally in 2019 to 152.8 million cases in 2050 (25). This “silent pandemic” will affect the informal patient support networks, mostly spouses and adult children, who provide a significant portion of the daily care needed in AD/ADRD cases (26).

NDIG in the context of AD/ADRD family caregiving is the caregiver's emotional and physical response to the perceived losses in a valued care recipient. Family caregivers experience a variety of emotions that can fluctuate over the course of a disease, from early diagnosis to the end of life (27). This NDIG is due to (a) care recipient psychological “death,” which is asynchronous with physical death; (b) a drawn-out and uncertain disease trajectory; (c) communication difficulties between the affected patient and the caregivers; and (d) deterioration in relationship quality, family roles and signification limitations in caregiver freedom (27, 28).

NDIG in the context of AD/ADRD caregiving has been found to be a psychological phenomenon with unique characteristics (29), and is a significant risk factor for depression, anxiety, and PGD. For example, a recently published systematic review (30) reported that studies documented that 17% of AD/ADRD caregivers met the PGD criteria pre-death, and 6–26% of participants met the complicated grief criteria post-death.

As mentioned, the focus on AD/ADRD constitutes a specific example, when there is considerable evidence of NDIG also among people whose loved-ones suffer from terminal cancer (31), persistent disturbance of consciousness (32, 33), brain injuries (34–36), mental illnesses (37), and so on.

## Discussion

In light of the extensive empirical evidence of the existence of the NDIG phenomenon, its scope and its potential consequences, there is great importance in deepening the professional discourse around the issue of the inclusion of an appropriate diagnostic category for maladaptive reactions. Nonetheless, a move to offer a specific and unique diagnosis that defines maladaptive responses in the NDIG processes needs to be balanced with a consideration of the potential risks and possible benefits that might follow from such a move.

First and foremost, despite the extensive existence of this grief, the vast majority of situations in which people experience NDIG falls within the range of ultimately adaptive and normative responses. It is a minority who experience disordered NDIG in a way that causes significant distress and functional impairment. This is similar to most existing diagnoses, such as reactions of anxiety, depression, and grief among the general population that do not meet the threshold for clinical diagnosis.

As is often reiterated, such extension of diagnostic categories in ultimately adaptive responses to loss raises concerns about pathologizing some cases within the domain of normative phenomena. Categorizing and labeling basically healthy people as suffering from a psychiatric disorder may lead to inflation in the assignment of diagnoses and various negative outcomes including over-prescription of medication. In addition, some may argue that there is not enough evidence to support the promotion of this discussion. Furthermore, the multitude of concepts and the lack of homogeneity in defining the grieving process may be a significant barrier as well.

Nevertheless, the empirical evidence that NDIG can lead to prolonged suffering with serious health consequences for the patients and their family members cannot be ignored. Therefore, the ultimate goal of the inclusion of a diagnosis of disordered NDIG is social and clinical recognition of the presence of symptoms of complex grief reactions, thus advancing the development of evidence-based assessment and intervention methods in order to alleviate the enduring emotional pain and improve the quality of life of people coping with high levels of NDIG.

The development and specification of well-defined criteria for the diagnosis is important to limit the concerns expressed above. And yet, even a circumscribed and limited initial diagnostic category would invite a broader healthcare awareness of the challenges faced by grieving relatives experiencing NDIG. Indeed, proceeding in this direction is certain to be controversial. However, in light of the considerable evidence collected in the last decades documenting these phenomena, their scope and consequences, we believe that advancing the discussion will raise awareness among healthcare professionals and society as a whole. A consequence of this awareness is providing greater legitimacy and social support for people experiencing NDIG, as well as facilitating the early identification of people at increased risk for maladaptive grief and adjustment difficulties. With early identification comes the ability to provide appropriate treatment and reduce suffering. Nevertheless, the significant ambiguity due to the different definitions of the NDIG concept constitutes a significant barrier that must be clarified in future discussion.

We have outlined preliminary arguments on the merits and concerns of diagnosing disordered NDIG. We are not implying that diagnostic criteria are in place. Discussions are needed on further conceptualizing and defining this spectrum of disorder, potential diagnostic criteria, the necessary and relevant circumstances of its occurrence, and its applicability across a range of terminal vs. chronic conditions. Only after these issues have been clarified will it be appropriate to consider possible placement in official manuals (e.g., as a unique diagnosis or a subtype of PGD). Reflecting on the evolution and history of the PGD diagnosis suggests that consensus-building around other grief disorders face an uncertain outcome. To comprehensively examine NDIG, there is a need to expand the professional literatures. By doing so, we can better discern ultimately adaptive from maladaptive responses of the grief characteristic of these losses. Recognizing the reasoning behind a potential diagnosis is vital to advance and understand the range of grief responses in non-death contexts. By raising this issue, we seek to advance a dialogue that facilitates theoretical, research, and therapeutic advancements, aiming to support individuals who are coping with these “transparent” and disenfranchised losses.

## Author contributions

AM: Conceptualization, Writing—original draft, Writing—review & editing. EY: Conceptualization, Writing—original draft, Writing—review & editing. SR: Conceptualization, Writing—original draft, Writing—review & editing.
